# Highly pathogenic avian influenza H5N1 virus outbreak among Cape cormorants (*Phalacrocorax capensis*) in Namibia, 2022

**DOI:** 10.1080/22221751.2023.2167610

**Published:** 2023-03-01

**Authors:** Umberto Molini, John Yabe, Irene K. Meki, Hatem Ouled Ahmed Ben Ali, Tirumala B. K. Settypalli, Sneha Datta, Lauren Michelle Coetzee, Ellini Hamunyela, Siegfried Khaiseb, Giovanni Cattoli, Charles E. Lamien, William G. Dundon

**Affiliations:** a School of Veterinary Medicine, Faculty of Health Sciences and Veterinary Medicine, University of Namibia, Windhoek, Namibia; b Central Veterinary Laboratory (CVL), Windhoek, Namibia; c Animal Production and Health Laboratory, Animal Production and Health Section, Department of Nuclear Sciences and Applications, Joint FAO/IAEA Division, International Atomic Energy Agency, Vienna, Austria

**Keywords:** Avian influenza, Namibia, Cape cormorant, H5N1, Clade 1.3.4.4b

## Abstract

In January 2022, significant mortality was observed among Cape cormorants (*Phalacrocorax capensis*) on the west coast of Namibia. Samples collected were shown to be positive for H5N1 avian influenza by multiplex RT-qPCR. Full genome analysis and phylogenetic analysis identified the viruses as belonging to clade 2.3.4.4b and that it clustered with similar viruses identified in Lesotho and Botswana in 2021. This is the first genomic characterization of H5N1 viruses in Namibia and has important implications for poultry disease management and wildlife conservation in the region.

Highly pathogenic avian influenza (HPAI) of the H5 subtype continues to be a threat to the global poultry industry and has caused significant economic losses in many countries in 2021–2022 [1–3]. The Gs/Gd HPAI H5NX lineage was first identified in Guandong province, China in 1996, and since then has diversified into ten clades based on the haemagglutinin (HA) gene. Of these clades, strains from Clade 2.3.4.4b have been the most recently detected in avian populations globally, both wild and domestic. In 2021 there were several reports of HPAI outbreaks in southern Africa (i.e. Botswana, Lesotho, South Africa) in both wild and domestic birds made to the World Organization for Animal Health (WOAH). The full genomes of the H5N1 Clade 2.3.4.4b viruses identified in Botswana and Lesotho have been published [4,5]. This current report describes the characterization and full genome sequences of an H5N1 Clade 2.3.4.4b from a Cape cormorant in Namibia.

In January 2022, more than 6500 Cape cormorant carcasses were retrieved from Bird Island, Walvis Bay, Erongo Region, Namibia (GPS: S 22°52’40.858″; E 14°32’8.446″). Bird Island is a man-made platform off the coast that serves as a breeding ground primarily for Cape cormorants and is a source of guano, which is collected and exported to South Africa where it is used as a component of garden fertiliser. No unusual mortality in other bird species was observed on, or near, the island. Retrospective examination of carcasses collected on the island showed that this mortality event began at the beginning of December 2021. An official report was made to the World Organization for Animal Health (WOAH) by Namibian authorities. The carcasses of three adult Cape cormorants (two males and one female) were collected, refrigerated, and sent to the Central Veterinary Laboratory (CVL) Windhoek, Namibia for necropsy and histopathological examination on the 30/01/2022.

A pool of organs for each of the cormorants (e.g. liver, lung, trachea and intestine) were homogenized in 1 mL of sterile phosphate-buffered saline using a TissueLyser LT (Qiagen, Hilden, Germany). RNA was extracted from 200 µL of the suspension using a commercial kit (High Pure Viral Nucleic Acid Kit, Roche, Basel, Switzerland) and resuspended in an elution volume of 100 µL. The matrix protein (M) gene of influenza A virus was detected by reverse transcription quantitative PCR (RT-qPCR) using a commercial kit (Genesig Advanced Kit Influenza A Virus (M1), Primerdesign Ltd., Southampton, UK). Purified RNA was sent to the Animal Health and Production Laboratory (APHL), Austria for confirmation and full genome sequence generation. On arrival at APHL, H5N1 was confirmed for all samples using a duplex RT-qPCR [6].

The purified RNA of one sample (A/Cormorant/Namibia/141/2022) was amplified according to the protocol of Zhou et al. [7] and the full genome was sequenced according to Makalo et al. [4]. The sequences have been submitted to GenBank under accession numbers OP776799 to OP776806.

Phylogenetic analysis using the HA gene of A/Cormorant/Namibia/141/2022 and other H5NX viruses retrieved from GenBank and GISAID demonstrated that the virus belonged to Clade 2.3.4.4b and that it was highly similar to H5N1 viruses identified in chickens in Lesotho in May 2021 and poultry and wild birds in Botswana in June 2021 [4,5] (Figure 1). Interestingly, the HA nucleotide sequence of A/Cormorant/Namibia/141/2022 was more similar at the nucleotide level to viruses identified in poultry in both Botswana (99.23%) and Lesotho (98.87%) compared to viruses in wilds birds (i.e. 98.26–98.50% in fish eagle and dove, respectively). Also of note was that the HA gene of A/Cormorant/Namibia/141/2022 was similarly identical (98.87%) to an H5N1 virus identified in a great white pelican (*Pelecanus onocrotalus*) in Senegal in 2021 but less so (96.5–98.38%) to other H5NX viruses from West Africa. Burkina Faso, Nigeria, Niger, and Senegal have seen a number of different HPAI outbreaks since 2021 in both poultry and wild birds. The regions wetlands are overwintering grounds for large numbers of migratory birds some of which then travel to southern Africa [8].

**Figure 1.**
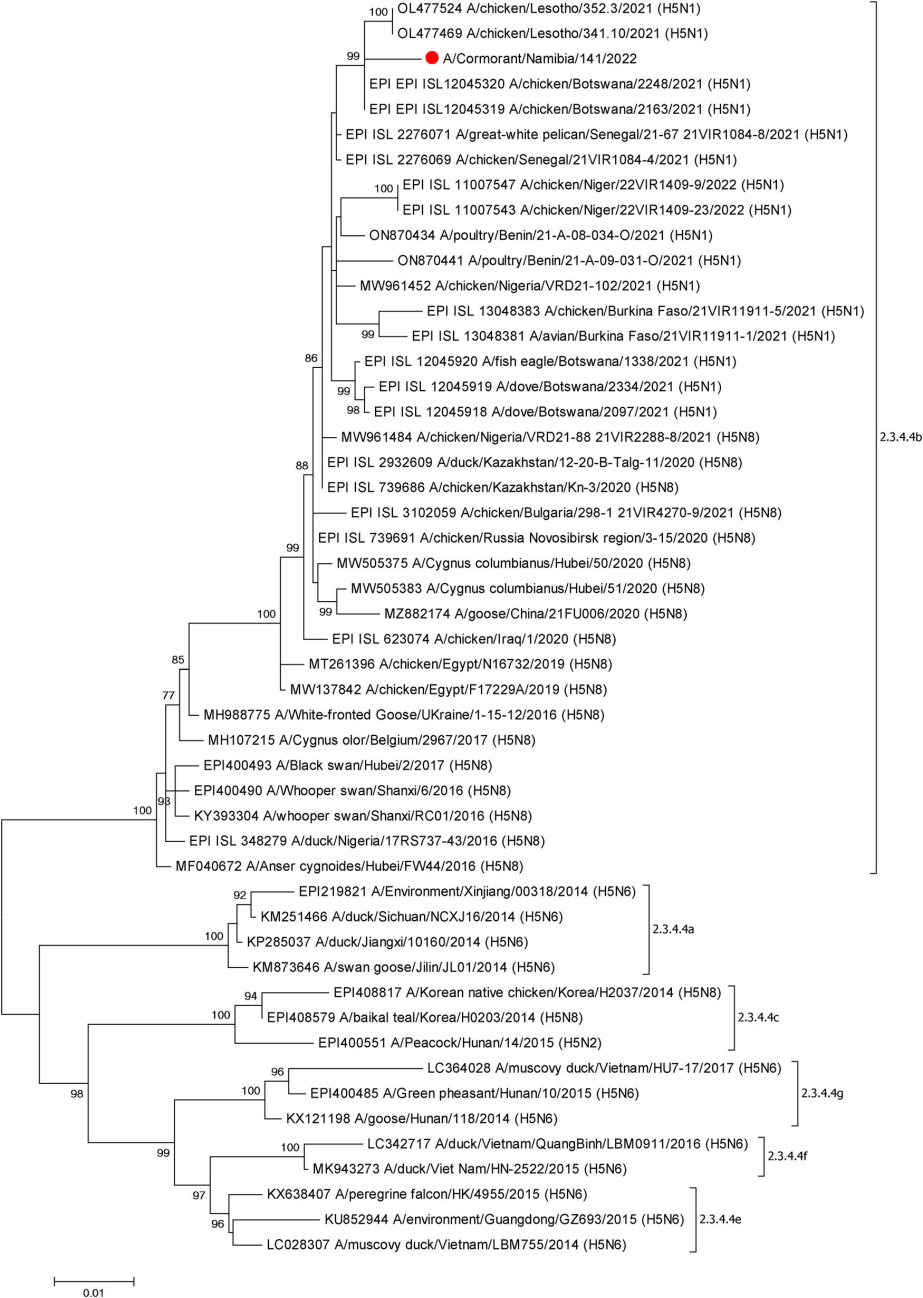
ML phylogenetic tree employing the Tamura-Nei model of nucleotide substitution with gamma distributed [with invariant sites (G + I)] rates among sites and 1000 bootstrap replications of the complete HA gene sequence (1704bp) from A/Cormorant/Namibia/141/2022 (red dot) combined with related sequences available in GenBank and GISAID. The sequences from this study are shown by filled red circles. Different subclades of clade 2.3.4.4 are also indicated.

Analysis of the HA amino acid sequence revealed a PLREKRRKRGLF highly pathogenic cleavage site and the presence of an QRG motif at position 222–224 of the receptor-binding site indicating a preference for α2-3 avian-like receptors. The ORF of the neuraminidase (NA) gene was 1410 bp in length and encoded of 469 amino acids. Stalk deletions in the NA, varying in size from 15 to 30 aa have been associated with viral transmission from wild bird to domestic poultry [9]. As no stalk deletion was identified in A/Cormorant/Namibia/141/2022, this would suggest that the virus originated from another wild bird or poultry in which virus circulation had been limited.

Pairwise analysis of the nucleotide sequence of the remaining genome segments [i.e. M, Nucleoprotein (NP), nuclear export protein (NEP), non-structural 1 proteins (NS1) polymerase basic protein 1 (PB1) and PB1-F2, polymerase basic protein 2 (PB2) and PA-X], revealed high identities (99.2–99.6%) with the viruses from Lesotho and Botswana and less so with viruses from West Africa. Recently, Clade 2.3.4.4b H5N1 viruses identified in outbreaks in mixed poultry farms in Burkina Faso in December 2021 were shown to be reassortants possessing a PA originating from an H9N2 AIV [10]. However, the PA from A/Cormorant/Namibia/141/2022 was >99% identical to the PA from the viruses from Lesotho and Botswana and, therefore, the virus is not a reassortant.

The PB2 had a glutamic acid (E) at position 627 indicating the lack of a mammalian adaptation motif identified by Herfst et al (2012) from studies in ferrets [11]. In addition, two unique mutations were identified in PB2 (i.e. E6K and C239G). Two mutations present in the NS1 protein (i.e. P42S and 103F) have both been reported to increase virulence of H5N1 viruses in mice, while unique amino acids in the M1 protein (i.e. L99M) and PB1 (i.e. V664M) were also identified.

The Cape cormorant is an endangered bird endemic to the southwestern coasts of Africa. Its breeding grounds extend from southern Namibia to the Western Cape of South Africa, while during the nonbreeding season the bird has also been found in northern Angola and Mozambique. Cape cormorants have previously been reported to be susceptible to HPAI from studies in South Africa [12]. The source of the virus infecting the Cape cormorants is unclear. Contact with other infected wild birds would be the most plausible explanation, although the phylogenetic analysis and comparison with wild bird and poultry viruses in Botswana and Lesotho would appear to contradict this. However, the number of samples characterised in these three countries is very limited and does not allow for definitive conclusions on the source of the virus. Nevertheless, other seabirds in the region known to be susceptible to AIV infection and which share the same environments with the Cape cormorant could have been potential sources. These include Swift terns (*Thalasseus bergii*), Sandwich Terns (*Thalasseus sandvicensis*), Common tern (*Sterna hirundo*), Hartlaub’s Gulls (*Chroicocephalus hartlaubii*) Cape Gannets (*Morus capensis*), African Oystercatchers (*Haemtopus moquini*) African penguins (*Spheniscus demersus*), and Crowned cormorants (*Microcarbo coronatus*) [12, 13].

This is not the first time that AIV has caused significant mortality among an endangered bird species in Namibia. In January 2019, a colony of African penguin were infected with a Clade 2.3.4.4b H5N8 [13]. As proposed by Peyrot et al. [12] the origin of this virus was most likely found in coastal seabirds from South Africa (2017–2018). Something similar appears to have occurred within the colony of endangered Cape cormorants which has been significantly reduced as a result.

In conclusion, the data provided for this study has important implications for both wild bird conservation and disease management in poultry. The results should encourage continuous monitoring of these bird communities by relevant authorities in Namibia and the region.
